# The burden of household out-of-pocket health expenditures in Ethiopia: estimates from a nationally representative survey (2015–16)

**DOI:** 10.1093/heapol/czaa044

**Published:** 2020-08-09

**Authors:** Mizan Kiros, Ermias Dessie, Abdulrahman Jbaily, Mieraf Taddesse Tolla, Kjell Arne Johansson, Ole F Norheim, Solomon Tessema Memirie, Stéphane Verguet

**Affiliations:** c1 Ethiopia Health Insurance Agency, Addis Ababa, Ethiopia; c2 Federal Ministry of Health, Addis Ababa, Ethiopia; c3 World Health Organization, Addis Ababa, Ethiopia; c4 Department of Global Health and Population, Harvard T.H. Chan School of Public Health, Boston, MA, USA; c5 Department of Global Public Health and Primary Care, University of Bergen, Bergen, Norway; c6 Department of Pediatrics and Child Health, College of Health Sciences, Addis Ababa University, Addis Ababa, Ethiopia

**Keywords:** Out-of-pocket expenditures, health expenditures, catastrophic expenditures, impoverishment, impoverishing expenditures, financial risk protection, equity, universal health coverage, Ethiopia

## Abstract

In Ethiopia, little is known about the extent of out-of-pocket health expenditures and the associated financial hardships at national and regional levels. We estimated the incidence of both catastrophic and impoverishing health expenditures using data from the 2015/16 Ethiopian household consumption and expenditure and welfare monitoring surveys. We computed incidence of catastrophic health expenditures (CHE) at 10% and 25% thresholds of total household consumption and 40% threshold of household capacity to pay, and impoverishing health expenditures (IHE) using Ethiopia's national poverty line (ETB 7184 per adult per year). Around 2.1% (SE: 0.2, *P* < 0.001) of households would face CHE with a 10% threshold of total consumption, and 0.9% (SE: 0.1, *P* < 0.001) of households would encounter IHE, annually in Ethiopia. CHE rates were high in the regions of Afar (5.8%, SE: 1.0, *P* < 0.001) and Benshangul-Gumuz (4.0%, SE: 0.8, *P* < 0.001). Oromia (*n* = 902 000), Amhara (*n* = 275 000) and Southern Nations Nationalities and Peoples (SNNP) (*n* = 268 000) regions would have the largest numbers of affected households, due to large population size. The IHE rates would also show similar patterns: high rates in Afar (5.0%, SE: 0.96, *P* < 0.001), Oromia (1.1%, SE: 0.22, *P* < 0.001) and Benshangul-Gumuz (0.9%, SE: 0.4, *P* = 0.02); a large number of households would be impoverished in Oromia (*n* = 356 000) and Amhara (*n* = 202 000) regions. In summary, a large number of households is facing financial hardship in Ethiopia, particularly in Afar, Benshangul-Gumuz, Oromia, Amhara and SNNP regions and this number would likely increase with greater health services utilization. We recommend regional-level analyses on services coverage to be conducted as some of the estimated low CHE/IHE regional values might be due to low services coverage. Periodic analyses on the financial hardship status of households could also be monitored to infer progress towards universal health coverage.

**Key Messages**

- In Ethiopia, ensuring financial risk protection in health is among the highest government priorities. However, little is known about the extent of out-of-pocket health expenditures and the associated financial hardships among Ethiopian households at national and regional levels.
- Our study shows that a large fraction of Ethiopian households faces financial hardship when accessing health services, particularly in Afar, Benshangul-Gumuz, Oromia, Amhara and SNNP regions.
- Periodic evaluation of financial risk protection should be conducted over time to identify progress towards UHC in Ethiopia, nationally and subnationally.

## Introduction

The primary goal of a health system is to improve the health of its population (Murray and Frenk, 2000; Roberts *et al.*, 2008). In order to achieve this goal, measures should be put in place to protect individuals and households from health-related expenditures that could impact their ability to access health care and their financial stability. Such actions are often referred to as financial risk protection (FRP) measures (Murray and Frenk, 2000; Roberts *et al.*, 2008).

The way governments collect revenue, pool risk and pay for health services eventually determines provision of FRP in a country (Gottret and Schieber, 2006; World Health Organization, World Bank, 2017). Provision of FRP implies that financial contributions towards health are determined by the capacity of households to pay and that access to health services is provided on the basis of needs (World Health Organization, 2010; World Health Organization, World Bank, 2017). This requires that national health systems mobilize adequate financial resources and do not rely too heavily on direct out-of-pocket (OOP) payments (Gottret and Schieber, 2006; Xu *et al.*, 2007; World Health Organization, 2010).

OOP health payments—what individuals would pay for health services (including medical and non-medical costs)—can impact the economic welfare of vulnerable households (World Health Organization, 2010). OOP payments are often used to finance and subsidize healthcare expenditures, which can sometimes place a significant financial burden on households. For example, this can force households to choose between paying for health care or basic needs such as food, housing and education (Wagstaff, 2009). When the costs of health services exceed households’ ability to pay, seeking care may be delayed or even avoided (Wagstaff, 2009; World Health Organization, 2010). Alternatively, if care is sought, ‘catastrophic’ health expenditures (CHE) could ensue; in some severe instances, OOP payments could also lead to poverty (Wagstaff and Doorslaer, 2003; Wagstaff, 2009). Cases of CHE are counted when OOP health expenditures exceed a certain threshold of household consumption expenditures or income (Wagstaff, 2009; Wagstaff *et al.*, 2018), such as 10% or 25% for example.

Globally, it was estimated that close to 800 million people would face CHE (at the threshold of OOP payments exceeding 10% of total household expenditure) in 2010 (World Health Organization, World Bank, 2017). A recent study reporting on CHE estimates in 133 countries demonstrated that CHE incidence had increased in almost half of the surveyed countries over the last decade (Wagstaff *et al.*, 2018). Similarly, as of 2010, globally, roughly 100 million people were estimated to be pushed under the international poverty line ($1.90 per day per capita, Purchasing Power Parity), and hence were likely to face impoverishment due to health spending (or so-called impoverishing health expenditures (IHE)) (World Health Organization, World Bank, 2017).

In Ethiopia, a low-income country with the second largest population in Africa (World Bank, 2019), the country’s Health Sector Transformation Plan (HSTP) and healthcare financing strategy envision reaching universal health coverage (UHC) by 2035 through the strengthening of primary healthcare services (Federal Democratic Republic of Ethiopia Ministry of Health, 2015, 2017a). Raising sufficient domestic finances, making service fees affordable, expanding the provision of exempted services, developing prepayment mechanisms and strengthening coverage for the poor are currently planned initiatives to improve FRP among Ethiopians (Federal Democratic Republic of Ethiopia Ministry of Health, 2015, 2017a). As a case in point, the Ethiopian government introduced community-based health insurance (CBHI) in 2011, covering over 25% of all districts by 2017, with a majority of those districts having established functional CBHI schemes enrolling nearly 3.5 million members, and >700 000 members being covered through government support (Federal Democratic Republic of Ethiopia Ministry of Health, 2017b). In addition, the government has exempted (i.e. fully covered the costs of) services related to high priority diseases and conditions like tuberculosis (TB), HIV and malaria, and maternal and child health (Federal Democratic Republic of Ethiopia Ministry of Health, 2017a).

According to the 2013/14 National Health Account (NHA) report, OOP health payments constituted about 33% of total health spending in Ethiopia (Federal Democratic Republic of Ethiopia Ministry of Health, 2017c), and such payments were particularly challenging for the 24% of the country’s population that live in extreme poverty (Federal Democratic Republic of Ethiopia National Planning Commission, 2017). About 73% of individuals who utilized health services would incur OOP payments, with 70% of those payments spent on medical costs (e.g. drugs, diagnostics) and 23% on non-medical costs (e.g. transportation, accommodation, food). Concerning sources for OOP spending, almost 55% would come from individuals’ or families’ own cash on-hand, whereas 35% would come from friends or relatives. Furthermore, about 18% of those needing health services were not able to access them due to financial barriers (Federal Democratic Republic of Ethiopia Ministry of Health, 2017c).

A CBHI pilot evaluation conducted in 13 districts of Oromia, Amhara, Tigray and Southern Nations Nationalities and Peoples (SNNP) regions indicated that about 3% of CBHI members and 9% of non-members incurred CHE (with a threshold of 25% of non-food expenditures) (Ethiopian Health Insurance Agency, 2015). Likewise, a facility-based study conducted in four major regions (Oromia, Amhara, SNNP and Tigray) and Addis Ababa City Administration on pneumonia and diarrhoea treatment for under-five children estimated impoverishment of 0.3% for outpatient pneumonia/diarrhoea episodes, and of 6–7% for severe pneumonia/diarrhoea episodes (with a poverty line of $1.25 per day) (Memirie *et al.*, 2017). Similarly, another study in selected hospitals of Addis Ababa estimated that about 27% of households seeking cardiovascular disease care would experience CHE (with a 10% threshold of annual income) (Tolla *et al.*, 2017).

Even though reduction in OOP payments and CHE incidence is a top priority for the Ethiopian government, to our knowledge, there exist no up-to-date subnational CHE and IHE estimates. Therefore, we generate in this article novel regional estimates on the incidence and distribution of CHE and IHE using Ethiopia’s national household consumption and expenditure (HCE) survey.

## Methods

We estimated the incidence of CHE and IHE among Ethiopian households at the national and regional levels.

### Data sources

We used secondary data from the 2015/16 Ethiopian household consumption and expenditure (HCE) and Welfare Monitoring (WM) surveys conducted by Ethiopia’s Central Statistical Agency (CSA). The HCE/WM surveys have been conducted every 4–5 years since 1995/96 to monitor the poverty situation in Ethiopia. The latest HCE/WM surveys, for the years 2015/16, covered 30 229 households (with a close to 100% response rate) and encompassed rural areas, major urban centres and medium- to small-sized towns (Federal Democratic Republic of Ethiopia Central Statistics Agency, 2016a). Both HCE and WM surveys used the same sample (i.e. the same households) to avoid any data inconsistency. The surveys were conducted over one full year, from July 2015 to July 2016, to control for all seasonal effects which might impact health services utilization and OOP health expenditures (Federal Democratic Republic of Ethiopia Central Statistics Agency, 2016a).

The list of households obtained from the 2007 population and housing census was used as a frame to select the sampled enumeration areas (EAs) in the country, where each EA consisted of about 150–200 households. To ensure accurate representation, multistage cluster sampling design was used (Federal Democratic Republic of Ethiopia Central Statistics Agency, 2016a).

Data were collected from each household using a standardized checklist with face-to-face interviews. The HCE survey checklist addressed basic household information including: demographic characteristics and economic activities, HCE data (on food, beverage, alcohol, tobacco, non-durable goods and frequent services) and household expenditure data on durable goods and less frequent services (e.g. clothing, footwear, dwelling rent, water, fuel energy, furniture, furnishing, household equipment, etc.) (Federal Democratic Republic of Ethiopia Central Statistics Agency, 2016a).

All direct OOP payments for formal and informal health services net of third-party payers (including fees for consultation, diagnostic tests, medicines, medical procedures, preventive health commodities, traditional medicine) and non-medical expenses (including transport, food and accommodation expenditures) were captured in the HCE survey and in our analysis.

Self-reported illness and health-seeking behaviour were incorporated in the WM survey (Federal Democratic Republic of Ethiopia Central Statistics Agency, 2016b), and individuals were asked about any illness experienced over the last 2 months preceding the survey, whether they had utilized care or not, and the associated amount of OOP payments incurred.

Access to HCE and WM survey secondary data was granted by Ethiopia’s CSA. De-identified anonymous datasets were used for the analysis.

### Data analysis

Data were cleaned and analysed using Stata (version IC 15) and ADePT (version 6) software (Wagstaff *et al.*, 2011). For each dataset (HCE and WM surveys), we generated a unique identification number for each household using a combination of variable codes per region, zone, district, EA and household. Then, we merged the HCE and WM surveys via the generated common household identification number, and checked for data completeness. The variables of interest used in our analysis were constructed based on the definitions provided by CSA (Federal Democratic Republic of Ethiopia Central Statistics Agency, 2016a) (see Supplementary Appendix Table S1 for further detail).

Health services utilization was calculated as the proportion of households with self-reported illness who sought care during the survey period (Federal Democratic Republic of Ethiopia Central Statistics Agency, 2016b). Individual-level consumption and expenditures were aggregated at the household level. To make households comparable in consumption and expenditures despite their differences in sex and age composition, we calculated the per adult equivalent consumption (Coudouel *et al.*, 2002; Wagstaff *et al.*, 2011) using Ethiopia’s Ministry of Finance and Economic Cooperation adult equivalent scale (see Supplementary Table S2 for further details). Furthermore, consumption was deflated using spatial and temporal price indices to accommodate for the differences in living standards among regions and time periods of data collection (Supplementary Tables S3 and S4).

The number of catastrophic headcounts (Hc) was used to measure CHE incidence. Hc represents the fraction of households whose OOP payments, as a share of income/total expenditures (budget share approach) or capacity-to-pay, would surpass a specific threshold denoted Y (Wagstaff, 2009). The threshold Y represents the point at which household OOP expenditures can impose a severe disruption to basic living conditions and the specific threshold value (10%, 25% or 40%) can vary when the denominator for calculating Hc is either total income/expenditures or capacity to pay (Wagstaff, 2009; World Health Organization, World Bank, 2017). In the base case scenario (Table 3), we used a threshold of 10% of total household consumption. Alternatively, for capacity to pay, it was defined under three scenarios: first, we deducted actual food expenditures from total expenditures; second, the food poverty line was deducted instead of the actual food spending for households whose spending was above the food poverty line; third, the national poverty line was deducted from the per adult equivalent household consumption (World Health Organization, World Bank, 2017) (see Supplementary Table S5).

Poverty headcount (denoted Hp) and poverty gap (denoted Gp) were used as measures of IHE. Hp estimates the number of households living below the poverty line as a percentage of all households, and Gp the poverty intensity (Wagstaff *et al.*, 2011). We further adjusted Gp to the international poverty line, as shown next.

Let *E_i_* be household *i’*s per adult consumption expenditure, *Z* be the poverty line and *N* be the sample size (i.e. the total number of households). We could then express:$\;Hp=1/N\times \sum_{i=1}^{N}B_{i}$, and$\;Gp=1/N\times \sum_{i=1}^{N}D_{i}$, where *D_i_* = *E_i_* – Z; and *B_i_* = 1 if *E_i_* < *Z* and 0 otherwise. A normalized *Gp* could be derived by dividing the average poverty gap of the population by the poverty line. Normalized mean positive *Gp* was calculated as the average poverty gap of the poor divided by the poverty headcount: N_*GP*_=Gp/Z, and$\;\mathrm{MN}{}_{\mathrm{Gp}}=Gp/Hp$. The poverty impact (PI) of IHE is then calculated by taking the difference between the pre-payment (pre) and post-payment (post) *Hp* and *Gp*: ${PI}^{Hp}={Hp}^{\mathrm{post}}{-Hp}^{\mathrm{pre}}$; $\;{PI}^{Gp}={Gp}^{\mathrm{post}}{-Gp}^{\mathrm{pre}}$;$\;{PI}^{N_{\mathrm{Gp}}}={N{}_{\mathrm{GP}}}^{\mathrm{post}}{-N{}_{\mathrm{GP}}}^{\mathrm{pre}}$; and ${PI}^{MN_{\mathrm{GP}}}={MN_{\mathrm{Gp}}}^{\mathrm{post}}{-MN_{\mathrm{Gp}}}^{\mathrm{pre}}$. The underlying methods implemented are detailed in Wagstaff and van Doorslaer (2003) and O'Donnell *et al.*, (2008).

The national poverty threshold (ETB 7184 per adult year, representing the cost of 2200 kcal per day per adult food consumption with an allowance for essential non-food items) (Federal Democratic Republic of Ethiopia National Planning Commission, 2017) was used to classify household living standards’ status (poor vs non-poor). Households were ranked in ascending order based on the real per adult equivalent total consumption expenditures, and divided into quintiles, with quintiles 1 (Q1) and 5 (Q5) representing the poorest and richest 20% of households, respectively. We could then estimate concentration indices to see if the percentage of households that experienced CHE was unequally distributed across the quintiles. Lastly, we tested the statistical significance of the results (*P* < 0.05).

## Results

The average national household size was 4.6 and the largest household size was in the Somali region (5.6 on average). About 19% of households lived in urban areas, whereas Harari (54%), Dire Dawa (65%) and Addis Ababa City administrations (100%) had high urban population proportions. About 73% of households were headed by men, and 46% of household heads were literate with the largest literacy rate being in Addis Ababa (89%). Overall, 13% of individuals reported having been ill over the last 2 months preceding the survey and the highest rate was in Gambella, Benshangul-Gumuz and Afar regions; 73% of those who reported being ill actually sought care with the highest rates being in Afar and Gambella regions (Table 1).

**Table 1:** Characteristics of surveyed households

Region	Mean HH size	Male HH head, in 1000s (%)	Urban HH, in 1000s (%)	Literate HH, in 1000s (%)^a^	Self- reported illness, in 1000s (%)	Health- seeking behavior, in 1000s (%)	Mean total consumption per adult equivalent (ETB)	Mean OOP per adult equivalent (ETB)	Share of OOP payments to total consumption (%)
Tigray	4.36	796 (67%)	1258 (24%)	2497 (56%)	865 (17%)	596 (69%)	14 018	144	1.06
Afar	4.61	245 (68%)	312 (19%)	516 (37%)	338 (20%)	305 (90%)	11 953	298	3.07
Amhara	4.09	3668 (73%)	3231 (16%)	8006 (44%)	3330 (16%)	2016 (61%)	12 051	115	1.00
Oromia	4.86	5312 (77%)	4779 (14%)	13 186 (46%)	4127 (12%)	3413 (83%)	12 060	197	1.58
Somali	5.61	609 (66%)	780 (15%)	1438 (33%)	321 (6%)	251 (78%)	9816	86	0.66
Benshangul- Gumuz	4.40	175 (76%)	200 (20%)	388 (45%)	269 (27%)	218 (81%)	13 307	217	2.16
SNNP	4.88	2742 (75%)	2704 (15%)	6646 (43%)	2262 (13%)	1860 (82%)	12 285	124	1.04
Gambella	4.62	57 (67%)	134 (34%)	195 (56%)	94 (24%)	83 (89%)	13 779	177	1.49
Harari	4.07	41 (71%)	130 (54%)	129 (63%)	39 (17%)	29 (76%)	18 419	277	1.41
Addis Ababa	3.90	473 (57%)	3243 (100%)	2627 (89%)	277 (9%)	231 (84%)	16 499	354	1.29
Dire Dawa	4.38	71 (72%)	282 (65%)	222 (58%)	79 (18%)	58 (74%)	16 193	226	1.10
Total	4.61	14 194 (73%)	17 058 (19%)	35 856 (46%)	12 005 (13%)	9065 (76%)	12 303	162	1.28

HH, household; SNNP, Southern Nations Nationalities and Peoples; ETB, Ethiopian Birr; OOP, out-of-pocket.

a Literate = able to read and write.

The mean consumption per adult equivalent was ETB12 300 (I$1557) annually, taking Purchasing Power Parity of 7.9 for 2015 (World Bank, 2020). The consumption was highest in Addis Ababa, Harari and Dire Dawa. The mean OOP payment per adult equivalent was ETB162 (I$20.5), where Addis Ababa, Afar and Harari had high OOP expenditures relative to the national average. The national share of OOP payments to household total budget was 1.3%, and Afar (3.1%) and Benshangul-Gumuz (2.2%) had relatively greater shares (Table 2).

**Table 2:** Distribution of self-reported illness and consumption, per consumption quintile in Ethiopia

Quintile	Share of self-reported illness (%)	Share of care seeking (%)	Consumption / adult equivalent (ETB)^a^	Share of consumption (%)	OOP/adult equivalent (ETB)	Share of OOP (%)	OOP/total consumption (%)
1	23	20	5 305	7.4	56	5.7	1.1
2	22	22	8774	12.3	102	10.5	1.2
3	20	20	11 401	16.0	146	15.1	1.3
4	19	19	15 583	21.9	205	21.1	1.4
5	16	18	30 255	42.4	461	47.6	1.5
National average	N/A	N/A	12 303		162		1.3
Gini coefficient			0.35*** (SE: 0.002)				
Concentration index					0.39 *** (SE: 0.01)		

a Consumption/adult equivalent, consumption adjusted for age and sex.

SE, standard error; OOP, out-of-pocket.

Significance level: *P < 0.05; **P < 0.01; ***P < 0.001.

Most of the basic household characteristics (Table 1) were consistent with other national estimates as reported by Ethiopia’s Demographic and Health Surveys, and National Health Accounts (Federal Democratic Republic of Ethiopia Central Statistics Agency, 2016c; Federal Democratic Republic of Ethiopia Ministry of Health, 2017c). The share of self-reported illness was slightly higher among the bottom consumption quintile while health-seeking behaviour was more or less similar across quintiles. OOP spending increased with income: the lowest and highest quintiles would capture 6% and 48% of total OOP spending, respectively.

**Figure 1:**
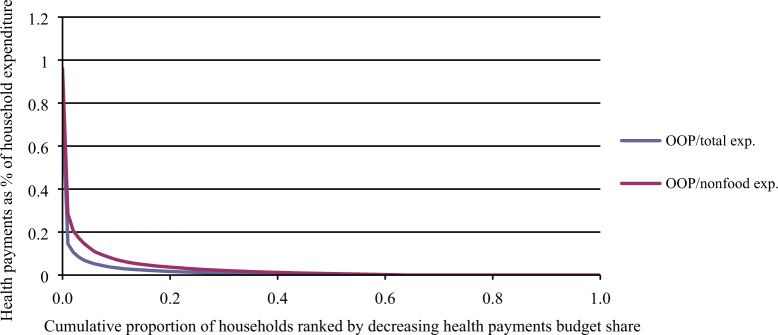
Cumulative distribution of OOP health expenditures in Ethiopia, as a ratio with either total expenditures or non-food expenditures.

Table 3 presents the estimated national and subnational CHE incidence (Hc) using a 10% threshold. The distribution of OOP payments with respect to income is displayed on Figure 1 (CHE estimates using capacity-to-pay thresholds provided in Supplementary Table S4). The national CHE rate was estimated at 2.1% (using a 10% threshold); and it was greater in Afar (5.8%), Benshangul-Gumuz (4.0%) and Harari (3.6%). However, given that Oromia, SNNP and Amhara regions have the largest populations, they would have the largest number of individuals with CHE in absolute terms (about 902 000, 268 000 and 275 000, respectively). The concentration index corresponding to Hc (using a 10% threshold) was 0.08 at the national level suggesting a rather low level of inequality. In addition, Table 4 summarizes the poverty headcount Hp using the national poverty line (ETB 7184 per adult per year): Hp would increase by 0.9 percentage points, from 23.8% to 24.7% after paying for health services; and the largest increase in Hp was estimated for Afar.

**Table 3:** Estimated incidence and distribution of CHE (with a 10% threshold of total household consumption), nationally and subnationally, in Ethiopia

	CHE		Concentration index	
	*N* (%)	SE (%)	Mean	SE
Region				
Tigray	97 672 (1.9) ***	0.4	0.15	0.09
Afar	97 131 (5.8) ***	1.0	0.04	0.08
Amhara	275 352 (1.3) ***	0.2	0.06	0.09
Oromia	901 681 (2.7) ***	0.3	0.08	0.07
Somali	58 560 (1.1) **	0.4	0.16	0.27
Benshangul-Gumuz	40 651 (4.0) ***	0.8	0.01	0.11
SNNP	267 778 (1.5) ***	0.3	0.01	0.10
Gambella	7850 (2.0)***	0.4	0.13	0.11
Harari	8590 (3.6) ***	0.8	−0.06	0.12
Addis Ababa	77 187 (2.4) ***	0.3	0.13*	0.07
Dire Dawa	8467 (1.9) **	0.7	0.11	0.21
National	1 840 919 (2.1) *	0.2	0.08*	0.04
Residence				
Urban	393 216 (2.3) ***	0.2	N/A	
Rural	1 447 703 (2.0) ***	0.2		

SE, standard error; SNNP, Southern Nations Nationalities and Peoples.

Significance level:

* *P* < 0.05;

** *P* < 0.01;

*** *P* < 0.001.

**Table 4:** Estimated poverty headcounts (Hp) in Ethiopia, at the national and regional levels

Poverty headcount (Hp)				
Region	Hp gross (%)	Hp net of health payments (%)	Net Hp, *N* (%)	SE (%)
Tigray	26.7	27.6	47 792 (0.9%) **	0.29
Afar	26.0	31.1	83 930 (5.0%) ***	0.96
Amhara	28.0	29.0	201 881 (1.0%) ***	0.24
Oromia	23.3	24.4	355 961 (1.1%) ***	0.22
Somali	23.3	23.4	2302 (0.1%)	0.03
Benshangul-Gumuz	27.2	28.1	9237 (0.9%)*	0.40
SNNP	20.7	21.0	54 301 (0.3%) **	0.11
Gambella	22.9	23.5	2198 (0.6%)*	0.25
Harari	9.1	9.1	0 (0.0%)	0.00
Addis Ababa	15.9	16.2	9272 (0.3%) **	0.09
Dire Dawa	15.4	16.7	5455 (1.3%)	0.74
Total	23.8	24.7	772 329 (0.9%) ***	0.11

SE, standard error.

Significance level:

* *P* < 0.05;

** *P* < 0.01;

*** *P* < 0.001.

**Figure 2:**
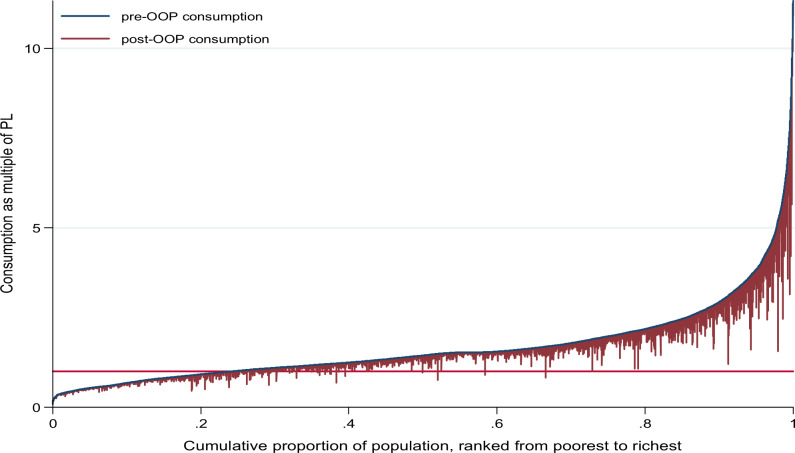
Effect of OOP health payments on household consumption expenditures. PL, poverty line (horizontal red line).

Figure 2 shows how household consumption changed before and after health services utilization. The vertical bar indicates the degree of reduction in consumption signalling household impoverishment when crossing the poverty line (horizontal red bar). Roughly, most of IHE would be observed among the bottom two quintiles. Lastly, Table 5 displays regional and national average poverty gaps (denoted Gp). The national average was estimated to increase by ETB16 per adult equivalent (0.2%) from ETB488 (6.8%) to ETB504 (7.0%) after OOP health payments. Among the poor, Gp was estimated to increase by 0.8% from the average shortfall of 28.5% (to 29.3%) after OOP payments. Similarly, the national depth of poverty increased from 6.8% of the poverty line to 7.0% after OOP payments suggesting that increase in poverty depth could result from many households becoming poor after OOP payments rather than the poor getting poorer. This was consistent across all regions, with impoverishment intensity being highest in Afar and Benshangul-Gumuz.

**Table 5:** Poverty depth estimated at the national and subnational levels in Ethiopia

	Poverty gap (Gp)^a^			Normalized poverty gap N_Gp_^b^				Mean normalized positive poverty gap MN_Gp_^c^			
Region	Gp gross	Gp net of health payments	Net Gp (mean and SE)	N_Gp_ gross (%)	N_Gp_ net of health payments (%)	Net N_Gp_ (%) (mean and SE)	N_Gp_ percentage change	MN_Gp_ gross (%)	MN_Gp_ net of health payments (%)	MN_Gp_ (%) (mean and SE)	MN_Gp_ percentage change
Tigray	506	518	13***(2.2)	7.0	7.2	0.18***(0.03)	2.55	26.40	26.90	0.51***(0.08)	1.92
Afar	331	386	56***(6.4)	4.6	5.4	0.77***(0.08)	16.80	17.68	19.97	2.29***(0.25)	12.95
Amhara	473	492	19***(3.0)	6.6	6.9	0.26***(0.04)	4.01	23.55	24.30	0.75***(0.13)	3.21
Oromia	482	501	19***(2.0)	6.7	7.0	0.26***(0.03)	3.85	28.80	29.71	0.92***(0.08)	3.18
Somali	669	676	7***(1.7)	9.3	9.4	0.1***(0.02)	1.07	39.88	40.29	0.41***(0.09)	1.03
Benshangul- Gumuz	407	447	40***(6.3)	5.7	6.2	0.56***(0.09)	9.90	20.81	22.81	1.99***(0.29)	9.58
SNNP	528	537	9***(1.3)	7.3	7.5	0.13***(0.02)	1.79	35.54	36.09	0.55***(0.06)	1.55
Gambella	416	434	18***(3.5)	5.8	6.0	0.25***(0.05)	4.25	25.28	26.29	1.01***(0.02)	4.01
Harari	251	259	7***(1.8)	3.5	3.6	0.1***(0.02)	2.94	38.62	39.76	1.14***(0.22)	2.94
Addis Ababa	268	279	11***(1.4)	3.7	3.9	0.16***(0.02)	4.26	23.47	24.30	0.84***(0.09)	3.57
Dire Dawa	265	276	12***(3.2)	3.7	3.8	0.16***(0.05)	4.35	23.91	24.66	0.75***(0.17)	3.12
National Average	488	504	16***(1.1)	6.8	7.0	0.23***(0.01)	3.38	28.54	29.33	0.79***(0.06)	2.78

a Poverty gap is calculated in monetary value by averaging out the difference between poverty line and per adult equivalent household consumption taking poverty gap zero for non-poor households.

b Normalized poverty gap is constructed by dividing poverty gap to poverty line for international comparison purpose. This metric avoids the effect of currency and difference in poverty line.

c Mean normalized positive poverty gap measures normalized poverty gap among the poor. Comparing the percentage change in mean normalized positive poverty gap to mean percentage change in normalized poverty gap will inform if the poor are getting poorer than the general households, as a result of OOP payments.

Significance level:

* *P* < 0.05;

** *P* < 0.01;

*** *P* < 0.001.

## Discussion

The objective of this study was to estimate the incidence and distribution of CHE and IHE at the national and regional levels in Ethiopia for the years 2015/16. Our analysis reveals that a substantial number of Ethiopian households would face financial hardship when accessing health services: around 2% and 1% of households would face CHE and IHE, respectively, with the highest rates being estimated for Afar and Benshangul-Gumuz regions.

Notably, Ethiopia’s estimates (CHE at 10% threshold: 0.8%, IHE: 0.4%) were lower compared with the sub-Saharan African regional averages (CHE: 10.3%, IHE: 1.6%), while having similar health services coverage index (about 40 out of 100), as reported by the 2017 UHC global monitoring report (World Health Organization, World Bank, 2017). This indicates that Ethiopia might present with relatively better FRP. In this respect, various studies in sub-Saharan Africa (e.g. Kenya, Nigeria, Tanzania) showed higher estimates of CHE incidence (7–18%, using a 10% threshold) than in Ethiopia, though direct comparisons remain difficult due to differences in sampling techniques, sample sizes, and types of analyses conducted (Chuma and Maina, 2012; Brinda *et al.*, 2014; Barasa *et al.*, 2017; Aregbeshola and Khan, 2018; Atake and Amendah, 2018).

The distribution of OOP payments estimated across income levels in our study seemed progressive but might also be related to differences in health services utilization or quality of care, as our estimates were a simple average per quintile and did not capture the number of visits nor care quality (and price) for the consumed OOP payments. There was low inequality in CHE incidence, with the richer households facing slightly higher CHE. The relatively higher rates of CHE/IHE in Afar and Benshangul-Gumuz could be explained by suboptimal implementation of exempted services, fee waivers and CBHI policies (Ethiopia Health Sector Financing Reform/Health Finance and Governance Project, 2018a). Surprisingly, IHE incidence was close to 0 in Addis Ababa City Administration and Harari region while having CHE above the national average. Low self-reported illness (Addis Ababa) and low poverty status to cope with high OOP payments (Harari and Addis Ababa) can be potential explanations. Good implementation of services exemption, fee waivers for the poor and CBHI in Amhara, Tigray and SNNP regions (Ethiopia Health Sector Financing Reform/Health Finance and Governance Project, 2018b, 2018c, 2018d) might explain the estimated lower rates of financial hardship in those regions. However, our findings should be interpreted with caution and should be complemented by regional-level effective coverage indices, as these estimated low CHE/IHE rates in our study could be well tied to low levels of health services utilization and/or lack of available services. The 2015/16 household services utilization and expenditure survey report showed low services utilization where outpatient attendance per capita was estimated to be at 0.56 (Federal Democratic Republic of Ethiopia Ministry of Health, 2017c).

Yet, our analysis presented a number of limitations. First, the surveys used did not capture the likely catastrophic indirect costs resulting from lower earnings and time losses following onset of illness. Households that did not seek care due to unaffordability were also not captured. Second, the various recall periods used for different goods and services, the valuing of home-produced goods and imputing of durable goods might create some noise to the data analysed. Third, due to the unclear classification of OOP expenditures by level of health care, service delivery and motive for payment—such as cost for consultation, drugs, lab tests, food, accommodation and transport—we could not address critical questions like the determinants of financial hardship.

In summary, the CHE and IHE in Ethiopia seemed to be lower than the sub-Saharan African average, but in absolute terms, large numbers of households are suffering from financial hardship in Ethiopia, especially in Afar, Benshangul-Gumuz, Oromia, Amhara and SNNP regions. Financial hardship could get worse in the future with improvements in health services coverage, as services are made available to the population without necessary appropriate public financing. We recommend further regional-level analyses on the UHC services coverage index to be conducted as some of the low CHE and IHE regional values estimated could be due to low services coverage rather than actual provision of FRP. In the future, expanding and strengthening fee waivers for the poor in non-CBHI districts, maintaining fee exemption for high priority services (e.g. depending on burden of disease and care need), and expanding prepayment mechanisms would likely help to further reduce the burden of medical impoverishment in Ethiopia. Periodic evaluation of FRP should thus be conducted over time to infer progress towards UHC in Ethiopia, nationally and subnationally. Such regular evaluations will enable the identification of health interventions to be publicly financed by the government so to maximize FRP gains per budget expenditure across disease categories (Verguet *et al.*, 2015, 2016).

## Supplementary data

Supplementary data are available at *Health Policy and Planning* online.

## Supplementary Material

czaa044_Supplementary_Data
