# Supplementary material for: The burden of household out-of-pocket health expenditures in Ethiopia: estimates from a nationally representative survey (2015–16)
Source: Health Policy Plan. 2020 Aug 9;35(8):1003–10. doi: 10.1093/heapol/czaa044 (PMC7553759; doi:10.1093/heapol/czaa044)
Supplement: czaa044_Supplementary_Data [file czaa044_supplementary_data.zip › czaa044-Suppl_Data/Supplementary_appendix_Track changes.docx]

**Supplementary appendix**

**Table S1.** Definition of variables used.

| **Definition** | **Source(s)** |
| --- | --- |
| **Household:**  A person or group of persons, whether or not they are related, who normally live together in the same housing unit or group of housing units, and who have common cooking arrangements. | [1] |
| **Member of household:**  A member of a household may be any of the following: (a) all persons who lived and ate with the household for at least six months (including those who were not present at the time of the survey but were expected to be absent from the household for less than six months); (b) all guests and visitors who ate and stayed with the household for six months or more; (c) housemaids, guards, babysitters, etc. who lived and ate with the household, even for less than six months. | [1] |
| **Household size**:  The total number of members of a household. | [1] |
| **Head of household:**  The person who economically supports or manages the household, or for reasons of age or respect, is considered as the head of the members of the household or otherwise declares him/herself as the head of a household. There may only be one head of household and this person may be male or female. | [1] |
| **Adult equivalent**:  All members of the household with adjusted calorie need requirement on the basis of age and sex. | Authors assumptions, on the basis of [1] |
| **Household consumption expenditures:**  They comprise both monetary and in-kind payment on all goods and services, and the monetary value of the consumption of home-made products. | [1] |
| **Food expenditures:**  Household food expenditures are the amount spent on food by the household plus the value of family’s own food productions consumed within the household. Excludes expenditures on alcoholic beverages and tobacco. | [1] |
| **Non-food expenditures:**  The difference in total household consumption expenditures and food expenditures. | [1] |
| **Out-of-pocket health expenditures**: Out-of-pocket health payments refer to the payments made by households when they receive health services net of third-party payments. Includes: fees for consultation, diagnostic tests, medicines, medical procedures, preventive health commodities, traditional medicine, and non-medical expenses (including transport, food and accommodation expenditures) | [1] |
| **Catastrophic headcounts:** Fraction of households whose OOP payments, as a share of income/total expenditures (budget share approach) or capacity-to-pay, would surpass a specific threshold denoted Y. The threshold Y represents the point at which household OOP expenditures can impose a severe disruption to basic living conditions and the specific threshold value (10, 25 or 40%) can vary when the denominator for calculating Hc is either total income/expenditures or capacity to pay. | Authors’ assumptions, on the basis of [2] |
| **Poverty line (poverty threshold):** Refers to the cost of 2,200 kcal per day per adult food consumption with an allowance for essential nonfood items. It is a line below which one is considered poor or not. | [3] |
| **Poverty headcount (Poverty headcount index):**  The share of the population whose income or consumption is below the national poverty line; that is, the share of the population that cannot afford to buy a basic basket of goods. | [3] |
| **Poverty gap (Poverty gap index):** Poverty gap provides information regarding how far households are from the poverty line (measures intensity of poverty). This measure captures the mean aggregate income or consumption shortfall relative to the poverty line across the whole population. | [3] |
| **Health seeking behavior:**  Proportion of households with self-reported illness during the survey period who sought care during that period. The recall period is two or twelve- months preceding the survey. | [4] |
| **Literacy:**  The ability to read and write a short message in any language for those aged 10 years and above. | [4] |

**Table S2**. Ethiopia’s Ministry of Finance and Economic Cooperationadult equivalent conversion factor for calorie analysis [4].

| **Age group (years)** | **Male** | **Female** |
| --- | --- | --- |
| < 1 | 0.30 | 0.30 |
| 1-2 | 0.46 | 0.46 |
| 2-3 | 0.54 | 0.54 |
| 3-5 | 0.62 | 0.62 |
| 5-7 | 0.74 | 0.70 |
| 7-10 | 0.84 | 0.72 |
| 10-12 | 0.88 | 0.78 |
| 12-14 | 0.96 | 0.84 |
| 14-16 | 1.06 | 0.86 |
| 16-18 | 1.14 | 0.86 |
| 18-30 | 1.04 | 0.80 |
| 30-60 | 1.00 | 0.82 |
| > 60 | 0.84 | 0.74 |

**Table S3.** Spatial price index of 2015/16 by reporting levels (national average=100) [5].

| **Reporting level** | **Food** | **Non-food** |
| --- | --- | --- |
| Tigray Rural | 1.010 | 0.783 |
| Mekele | 1.146 | 1.168 |
| Other Tigray Urban | 0.999 | 0.973 |
| Afar Rural | 0.927 | 0.804 |
| Asayta Town | 1.065 | 0.803 |
| Other Afar Urban | 1.138 | 0.989 |
| Amhara Rural | 0.895 | 0.762 |
| Bahir Dar | 0.894 | 1.113 |
| Gonder | 0.990 | 1.184 |
| Dessie | 1.031 | 1.193 |
| Debreberhan | 1.002 | 0.972 |
| Other Amhara Urban | 0.985 | 0.940 |
| Oromia Rural | 1.055 | 0.960 |
| DebreZeite | 1.031 | 1.105 |
| Jimma | 0.915 | 1.134 |
| Adama | 1.085 | 1.113 |
| Nekemte | 0.940 | 0.922 |
| Shasheme | 0.971 | 0.984 |
| Other Oromia Urban | 0.936 | 1.014 |
| Somali Rural | 1.184 | 1.333 |
| Jigjjga | 1.015 | 1.162 |
| Other Somali Urban | 1.668 | 0.936 |
| Benshangul Gumuz Rural | 0.780 | 0.760 |
| Assosa | 0.949 | 0.866 |
| Other Benshangul Urban | 0.902 | 0.901 |
| SNNP Rural | 0.804 | 0.997 |
| Hawassa | 1.050 | 1.028 |
| Hosaena | 1.010 | 0.890 |
| Dila_tow | 1.000 | 0.947 |
| Arba_min | 1.032 | 1.098 |
| Sodo | 1.050 | 0.903 |
| Other SNNP | 0.919 | 0.972 |
| Gambella Rural | 0.953 | 0.750 |
| Gambella | 1.057 | 0.958 |
| Other Gambella Urban | 1.003 | 0.949 |
| Harari Rural | 0.859 | 0.965 |
| Harari Urban | 1.009 | 1.381 |
| Addis Ababa | 1.235 | 1.616 |
| Dire Dawa Rural | 0.995 | 1.020 |
| Dire Dawa Urban | 1.054 | 1.283 |

**Table S4.** Temporal consumer price index at the country level (December 2011 = 100) [6].

| **Month and year** | **General index (December 2011=100)** | **Food and non- alcoholic beverage (December 2011=100)** | | **Non-food index (December 2011=100)** | | **General index** | | **Food and non- alcoholic beverage** | | **Non-food index** | |  |
| --- | --- | --- | --- | --- | --- | --- | --- | --- | --- | --- | --- | --- |
| *Dec-15* | 145.9 | | 145.5 | | 146.4 | | 1.00 | | 1.00 | | 1.00 | |
| *Jul-15* | 146.0 | | 147.8 | | 143.9 | | 1.00 | | 1.02 | | 0.98 | |
| *Aug-15* | 146.7 | | 149.5 | | 143.6 | | 1.01 | | 1.03 | | 0.98 | |
| *Sep-15* | 148.4 | | 152.3 | | 144.1 | | 1.02 | | 1.05 | | 0.98 | |
| *Oct-15* | 148.8 | | 152.7 | | 144.5 | | 1.02 | | 1.05 | | 0.99 | |
| *Nov-15* | 145.8 | | 145.7 | | 145.9 | | 1.00 | | 1.00 | | 1.00 | |
| *Dec-15* | 145.9 | | 145.5 | | 146.4 | | 1.00 | | 1.00 | | 1.00 | |
| *Jan-16* | 147.6 | | 146.6 | | 148.6 | | 1.01 | | 1.01 | | 1.02 | |
| *Feb-16* | 147.4 | | 144.6 | | 150.5 | | 1.01 | | 0.99 | | 1.03 | |
| *Mar-16* | 148.3 | | 146.2 | | 150.6 | | 1.02 | | 1.00 | | 1.03 | |
| *Apr-16* | 150.5 | | 148.0 | | 153.3 | | 1.03 | | 1.02 | | 1.05 | |
| *May-16* | 152.5 | | 151.9 | | 153.1 | | 1.05 | | 1.04 | | 1.05 | |
| *Jun-16* | 154.0 | | 153.8 | | 154.2 | | 1.06 | | 1.06 | | 1.05 | |
| *Average index*  *(Dec 2015=100)* |  | |  | |  | | 1.02 | | 1.02 | | 1.01 | |

**Table S5.** Proportion of households facing catastrophic health expenditures (CHE).

| **Region** | **CHE using total consumption expenditures as denominator (%)** | **CHE using capacity to pay as a denominator and 40% as a threshold (%)** | | |
| --- | --- | --- | --- | --- |
|  | CHE, 25%* | Scenario 1† | Scenario 2‡ | Scenario3** |
| Tigray | 0.01 | 0.19 | 0.12 | 15.22 |
| Afar | 0.26 | 0.51 | 0.29 | 35.03 |
| Amhara | 0.11 | 0.33 | 0.22 | 16.00 |
| Oromia | 0.03 | 0.63 | 0.43 | 16.64 |
| Somali | 0.00 | 0.12 | 0.04 | 7.57 |
| Benshangul-Gumuz | 0.00 | 0.73 | 0.33 | 26.02 |
| SNNP | 0.00 | 0.17 | 0.07 | 12.14 |
| Gambella | 0.05 | 0.19 | 0.05 | 14.02 |
| Harari | 0.00 | 0.20 | 0.00 | 7.69 |
| Addis Ababa | 0.14 | 0.55 | 0.35 | 11.09 |
| Dire Dawa | 0.00 | 0.09 | 0.00 | 11.36 |
| Total | 0.05 | 0.41 | 0.26 | 15.18 |
| *CHE with out-of-pocket payments greater than 25% of total household consumption as a threshold. | | | | |
| †Scenario 1 = using non-food expenditures as a denominator, i.e. food expenditures deducted from total household expenditures to generate non-food expenditures [7]. | | | | |
| ‡Scenario 2 = deducted food poverty line from per adult consumption of households if actual food expenditures surpass food poverty line of the household and for others, actual food spending is deducted like scenario 1 to construct the capacity to pay [7]. | | | | |
| **Scenario 3 = denominator calculated by deducting national poverty line from per adult consumption of household. This approach assumes the capacity to pay should be the consumption that remains after covering the basic necessities [7]. | | | | |

**References**

1. Federal Democratic Republic of Ethiopia Central Statistic Agency. Household Consumption-Expenditure (HCE) Survey. Addis Ababa: Federal Democratic Republic of Ethiopia Central Statistic Agency, 2016.
2. Wagstaff A. Measuring financial protection in health. In: Smith PC, Mossialos E, Papanicolas I, Leatherman S, editors. Performance measurement for health system improvement. Cambridge, UK: Cambridge University Press, 2009; pp.114-137.
3. Federal Democratic Republic of Ethiopia National Planning Commission. Ethiopia’s Progress Towards Eradicating Poverty: an interim report on 2015/16 poverty analysis study. Addis Ababa: Federal Democratic Republic of Ethiopia National Planning Commission, 2017.
4. Federal Democratic Republic of Ethiopia Central Statistic Agency. Welfare Monitoring Survey. Addis Ababa: Federal Democratic Republic of Ethiopia Central Statistic Agency, 2016.
5. Federal Democratic Republic of Ethiopia Central statistics Agency: Country and Regional level consumer price indices for the month of January 2016, information No.35.
6. World Health Organization and World Bank. Tracking universal health coverage: 2017 global monitoring report. Geneva: World Health Organization and World Bank; 2017.
